# Post-Alignment Adjustment and Its Automation

**DOI:** 10.3390/genes12111809

**Published:** 2021-11-18

**Authors:** Xuhua Xia

**Affiliations:** 1 Department of Biology, University of Ottawa, Marie-Curie Private, Ottawa, ON K1N 9A7, Canada; xxia@uottawa.ca; Tel.: +1-613-562-5718; 2 Ottawa Institute of Systems Biology, University of Ottawa, Ottawa, ON K1H 8M5, Canada

**Keywords:** sequence alignment, automation, sum-of-pairs score, inconsistency, position weight matrix, PWM, codon-based alignment, phylogenetics

## Abstract

Multiple sequence alignment (MSA) is the basis for almost all sequence comparison and molecular phylogenetic inferences. Large-scale genomic analyses are typically associated with automated progressive MSA without subsequent manual adjustment, which itself is often error-prone because of the lack of a consistent and explicit criterion. Here, I outlined several commonly encountered alignment errors that cannot be avoided by progressive MSA for nucleotide, amino acid, and codon sequences. Methods that could be automated to fix such alignment errors were then presented. I emphasized the utility of position weight matrix as a new tool for MSA refinement and illustrated its usage by refining the MSA of nucleotide and amino acid sequences. The main advantages of the position weight matrix approach include (1) its use of information from all sequences, in contrast to other commonly used methods based on pairwise alignment scores and inconsistency measures, and (2) its speedy computation, making it suitable for a large number of long viral genomic sequences.

## 1. Introduction

High-quality multiple sequence alignment (MSA) is crucially important in sequence comparison and molecular phylogenetics because a poor alignment typically leads to bias and inaccuracy in phylogenetic estimation [1,2,3]. This is especially true in the present day, where the availability of an increasing number of sequences of increasing sequence lengths is often associated with the application of quick-and-dirty options in sequence alignment programs. This has resulted in poor sequence alignments in publications, even in prominent journals ([4,5], pp. 16–21 in reference [5]), highlighting the extent of the issue.

MSA was traditionally followed by post-alignment visual inspection and manual adjustment. However, such post-alignment improvements gradually faded away because of three contributing factors. Firstly, it becomes less important with the emergence of more accurate MSA programs such as MUSCLE [6] and MAFFT [7] with multiple iterations of MSA refinement [7,8,9]. Secondly, MSA in the genomic era often involve thousands of long sequences, as was frequently performed in sequence comparison of SARS-CoV-2 genomes [10,11], rendering it impractical to perform manual adjustment. Thirdly, post-alignment adjustment can be error prone and inconsistent because there is no explicit and consistent criterion that is universally used by researchers.

While sequence alignment with dynamic programming is guaranteed to generate the optimal sequence alignment given a scoring scheme [5,12], or at least one of the equally optimal alignments, progressive MSA has always been used in practice, generating sequence alignment that may well be suboptimal. This is because what is suboptimal is often not obvious when aligning closely related sequences. Only when more sequences are added to the alignment can one observe the suboptimality in previous alignment [13]. Multiple iterations of new guide trees and new MSA cannot refine such suboptimal alignment.

**Figure 1:**
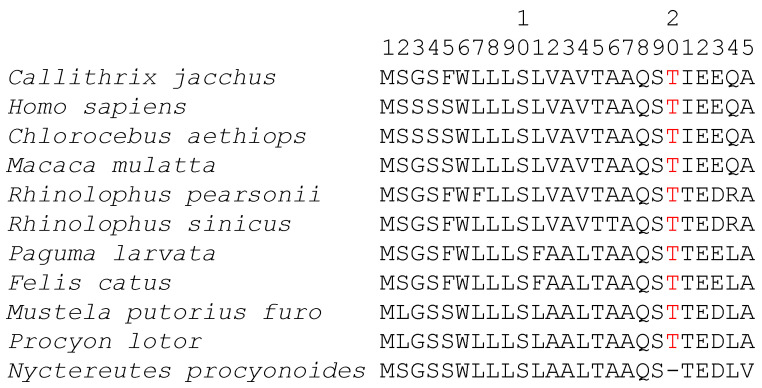
Multiple sequence alignment of 11 mammalian ACE2 proteins. Only 25 amino acid sites from the N-terminus are shown, taken from Wei et al. [14].

I illustrate two such cases. The first involves aligned sequences (Figure 1) taken from a study of mammalian ACE2 sequences in an effort to predict which mammalian species might be susceptible to SARS-CoV-2 infection [14]. The sequences were aligned with MAFFT with all optimization options selected. Only the first 25 amino acid sites are shown. We note that “-T” at sites 20 and 21 in *Nyctereutes procyonoides* should be “T-”. However, *N. procyonoides* is more closely related to *Procyon lotor* and *Mustela putorius furo,* so the three sequences will be aligned first in progressive MSA. “-T” and “T-” are equally good when aligning these three sequences, so one of the two equally good alignments needs to be chosen. MAFFT happens to choose “-T”, which turns out to be suboptimal when the first four sequences are added to the MSA.

**Figure 2:**
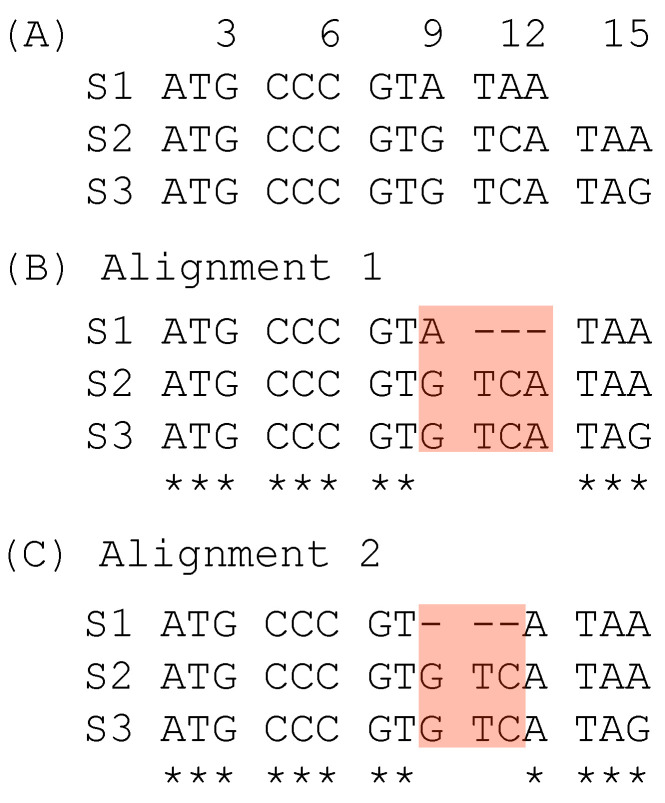
Suboptimal alignment of codon sequences. (**A**) Two unaligned codon sequences. (**B**) Alignment from codon-based alignment methods. (**C**) A better alignment based on alignment scores.

The second case of suboptimal alignment is caused not by the progressive MSA, but by codon sequence alignment which takes one of two approaches. One approach is to translate codon sequences into amino acid sequences, align the amino acid sequences, and then map the codons to aligned amino acid sequences ([15,16], pp. 38–39). The other approach is to align codon sequences directly with a 64 × 64 scoring matrix, as is implemented in the PhyPA function of DAMBE [17,18]. A simple illustration of a suboptimal alignment obtained with the codon-based alignment is shown with three codon sequences (Figure 2A). Alignment 1 (Figure 2B) is obtained from codon-based alignment. It contains one triplet deletion and an A ↔ G substitution. In contrast, Alignment 2 (Figure 2C) contains only a triplet deletion and consequently represents a simper hypothesis with a higher alignment score than Alignment 1. Alignment 2 can be obtained from Alignment 1 in post-alignment adjustment.

Such suboptimal alignments (Figure 1 and Figure 2) are not further refined by MSA programs such as the popular MUSCLE [6] and MAFFT [7]. However, one may formulate criteria to evaluate such suboptimal sites and make adjustment after the alignment. I present three methods based on three different criteria for this purpose: (1) sum-of-pairs score, (2) pairwise alignment inconsistency index, and (3) position weight matrix differential. The first two criteria are in general concordant, but they can conflict with the last criterion. However, the last one can often generate better MSA, leading to phylogenetic trees of higher likelihood than the other two criteria.

## 2. Criteria and Methods Used to Identify Suboptimal Sites in Alignments

Ideally, one would use maximum likelihood (ML) as a criterion for choosing the best alignment. From the same set of sequences, alternative alignment algorithmsa and scoring schemes may generate alternative alignments (MSA_1_, MSA_2_, …, MSA_n_). From each of MSAs, one may reconstruct a maximum likelihood tree (T_1_, T_2_, …, T_n_), with associated tree log-likelihood (lnL_1_, lnL_2_, …, lnL_n_). MSA_i_ is the best if lnL_i_ is the largest. This application of the ML criterion needs to be conditional on the number of gaps in each alignment because an alignment with many additional indels to minimize nucleotide or amino acid mismatches will tend to increase likelihood. However, the real difficulty with integrating both an MSA and a phylogeny in a ML criterion is that it would be too slow to be practical [19,20].

I present three practical criteria and associated approaches for post-alignment adjustment. The first two has been criticized for not making use of information in all sequences simultaneous or not considering the evolutionary history among the sequences [13,21]. The last does use information from all sequences simultaneously, although it still does not make use of the evolutionary history of the sequences. I hope that the approaches presented here will foster more innovative approaches.

### 2.1. Sum-of-Pairs Score (SPS)

Sum-of-pairs score (SPS) [22,23,24,25,26] has frequently been used as a criterion for evaluating alternative multiple alignment because of its conceptual simplicity. Each multiple alignment of *N* sequences implies *N*(*N*−1)/2 pairwise alignments. SPS is simply the summation of all pairwise alignment scores without penalizing shared gaps. Obtaining SPS from MSAs is easy. All we need is a scoring scheme, i.e., gap-open and gap-extension penalties plus a match/mismatch matrix. A slight variation of SPS is the weighted SPS [13,21] in which alignment scores for some sequence pairs are weighted more heavily than others. This is expressed as

(1) $$WSPS=\sum^{\;}W_{ij}S_{ij}$$

which is reduced to SPS when *W_ij_* = 1. MAFFT [7] uses multiple iterations of MSA refinement based on *WSPS* when either G-INS-i or L-INS-i option is chosen.

We need to evaluate Alignment 1 with “-T” (Figure 1) and Alignment 2 with “T-”, occupying sites 20 and 21 in the *N. procyonoides* sequence. We only need to compute SPS for these two sites. Suppose we use BLOSUM62 score matrix and a gap penalty of −6 as our scoring scheme. In this particular case, we only need to compute pairwise alignment scores between *N. procyonoides* and the other 10 species because all other pairwise scores are identical between the two alternative alignments (represented by constant C in Table 1).

For the 10 pairwise comparisons between *N. procyonoides* and the other 10 sequences at amino acid sites 20 and 21, Alignment 1 has 10 “T/-” pairs, 6 “T/T” pairs and 4 T/I pairs, yielding an SPS of −34 + C (Table 1). In contrast, Alignment 2 has 6 “T/-” pairs, 10 “T/T” pairs and 4 I/- pairs, yielding an SPS of −10 + C (Table 1). Therefore, Alignment 2 is better than Alignment 1.

The same approach can be applied to evaluate sites 9–12 in the two alternative alignments in Figure 2. Alignment 1 has both a triplet deletion and a nucleotide substitution, in contrast to Alignment 2 with only a triplet deletion but no nucleotide substitution, so SPS is greater for Alignment 2 than for Alignment 1.

### 2.2. Pairwise Alignment Inconsistency Index (PAI)

*N* sequences have *N*(*N*−1)/2 pairwise alignments. Pairwise alignments can be inconsistent with each other and with those implied by MSA, which had been used to refine MSA before [17,27,28,29,30,31]. Designating *S_ij_* as the pairwise alignment score between sequences *i* and *j*, and *S_ij.MSA_* as the equivalent score for paired alignment implied by the MSA, *PAI* is

(2) $$PAI=\sum^{\;}S_{ij}-\sum^{\;}S_{ij.MSA}$$

Because *S_ij_* is from dynamic programming and consequently has the highest possible alignment score, whereas *S_ij.MSA_* is from the pairwise alignment implied by the progressive MSA, *S_ij_* ≥ *S_ij.MSA_*. A poor MSA will have a larger *PAI* than a good MSA. For the 11 amino acid sequences (Figure 1) with Alignment 1 and Alignment 2 as defined before, we only need to compare the pairwise alignments between *N. procyonoides* and the other 10 sequences for sites 20 and 21. *PAI* for Alignment 1 is greater than *PAI* for Alignment 2 by a difference of 24. We conclude that Alignment 1 is worse than Alignment 2. The advantage of using *PAI* is that all *S_ij_* values are already computed in first guide tree during MSA, so there is little computational overhead.

### 2.3. Position Weight Matrix Differential (PWMD)

Position weight matrix (PWM) [32,33,34,35,36] was originally introduced into biology for characterizing regulatory motifs as components of regulons [37,38]. Its computation, as well as associated significance tests, has previously been illustrated numerically in great detail [37,39]. PWM scores (*PWMSs*) have been suggested as possible metrics for evaluating alternative MSAs [37]. A PWM can be generated from an MSA, and *PWMS* can be computed for each sequence. When one or more nucleotides or amino acids are shifted along indels, the difference in *PWMS* before and after the shifting is

(3) $$PWMD=\sum^{\;}PWMS_{after}-\sum^{\;}PWMS_{before}$$

Any nucleotide or amino acid shift that results in a positive *PWMD* is desirable. The 11 ACE2 sequences in Figure 1 have an alignment length of 805, so the resulting PWM is a 20 × 805 matrix. However, we only need to look at sites 20 and 21 (Table 2). Site 20 is occupied by amino acid T, so only amino acid T has a positive value at site 20. Site 21 is occupied by both T and I, so only these two amino acids have positive values at site 21 (Table 2). The PWM from either Alignment 1 or Alignment 2 suggest that we should put amino acid T at site 20 instead of at site 21 in *N. procyonoides.* For the PWM derived from Alignment 1, placing T at site 20 instead of 21 yields a PWMD of 0.6481 (=4.2457−3.5976, Table 2).

The previous presentation of the three approaches might mislead the reader to think that the two criteria are all consistent with each other. This is unfortunately not the case. While the first two approaches are generally consistent with each other, they often conflict with the third criterion (PWMD). I will illustrate this with a more realistic data set with alignment of huntingtin (HTT) proteins.

## 3. A comparison of Methods with Huntingtin Sequence Alignment

Huntington’s disease is associated with the length of glutamine (Q, encoded by CAG and CAA codons) repeats in the huntingtin (HTT) protein. The expansion and shrinking of (CAG)*_n_*, where the subscript *n* is the number of consecutive CAG codons, is caused by strand slippage during DNA replication [41]. Huntington’s disease typically manifests with *n* > 37. The longer the repeats, the earlier the disease onset [42].

**Figure 3:**
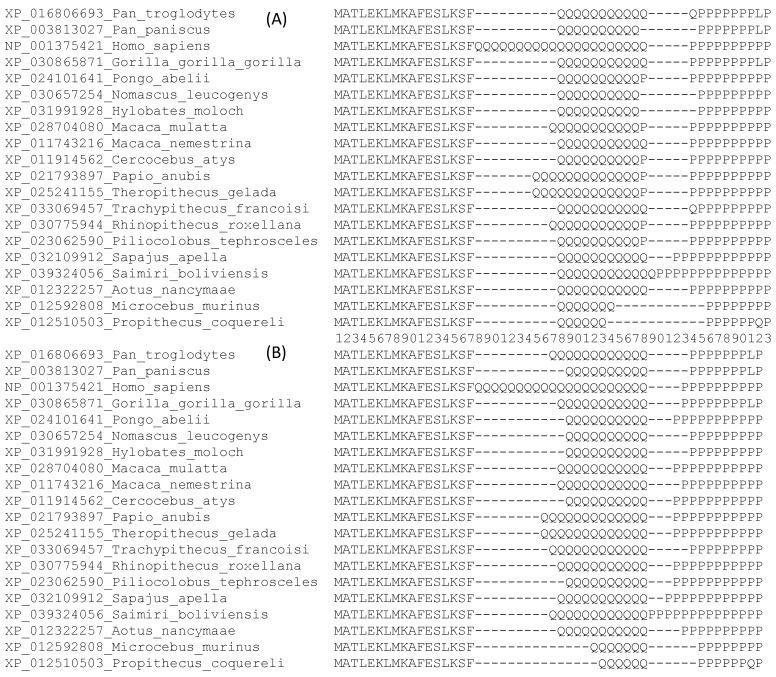
N-terminus of 20 aligned HTT sequences, with the site numbering in the middle. (**A**) Alignment from MAFFT [7] with optimized options. (**B**) One of the alternative alignments refined with the PWMD criterion.

I downloaded 20 primate HTT protein sequences and aligned them using MAFFT [7] with the slow but accurate G-INS-i option that uses progressive alignment with multiple iterative refinements based on weighted sum-of-pairs score as defined in Equation (1). The aligned sequences are included in FASTA format in the Supplemental file Primate_HTT_MAFFT.fas.zip. The MSA contains 3156 aligned sites, but only the first 53 sites are shown in Figure 3A for illustrating the PWM-based post-alignment refinement. This alignment is contrasted with an alternative alignment (Figure 3B), based on PWMD that I will explain later.

Both SPS and PAI indices would favor the alignment in Figure 3A against that in Figure 3B. For example, if we use a gap open (GO) penalty of 20, a gap extension penalty of 2, and the BLOSUM62 score matrix, then SPS is 31,007 for the MSA in Figure 3A, but only 29,178 for the MSA in Figure 3B. This is clearly seen from the pairwise alignment between the first (*Pan troglodytes*) and the third (*Homo sapiens*) sequences. There is only one GO in the alignment between these two sequences in Figure 3A but two GOs in Figure 3B. A number of similar differences contribute to a much larger SPS for the alignment in Figure 3A than that in Figure 3B. Therefore, the SPS and PAI indices would favor the alignment in Figure 3A against the alignment in Figure 3B.

A likelihood method, however, would favor the alignment in Figure 3B against that in Figure 3A. We may reconstruct a phylogenetic tree from each of these two alignments using PhyML [43] with (1) the LG substitution matrix and (2) a constant rate of amino acid substitution over sites. This yields a tree log-likelihood (lnL) of −126.69 for the alignment in Figure 3A but −106.77 for the alignment in Figure 3B. One may change substitution matrices but the tree lnL is consistently greater for the alignment in Figure 3B than for the alignment in Figure 3A (Table 3).

One might argue that the ML criterion in Table 3 is not fair because there are more amino acid substitutions in the alignment in Figure 3A than in Figure 3B. This is a valid criticism. However, one may defend the alignment in Figure 3B in two ways. First, the alignment in Figure 3B did not add indels to reduce amino acid substitutions. In fact, the alignment in Figure 3B has 20 fewer gaps than that in Figure 3A. Second, the alignment in Figure 3B suggests that the expansion/shrinkage of repeated amino acid Q occurs more frequently than amino acid replacement. This is consistent with the documented strand slippage during DNA replication in generating length variations of (CAG)*_n_* tracts in the *HTT* gene [41].

The alignment in Figure 3B is one of the optimal alignments based on the PWMD criterion. It highlights a case where the criterion of PWMD conflicts with SPS and PAI. I outline below the steps involved in post-alignment adjustment involving PWMD.

**Step 1:** From the alignment of HTT sequences obtained from MAFFT with 3156 aligned sites, one can compute the 20 × 3156 PWM. Part of the PWM, with the relevant sites and the two relevant amino acids (Q and P) is shown in Table 4. The PWM values in the “Q” column (Table 4) state that Q_6_ (where the subscript 6 is the number of consecutive Qs in a sequence) should be placed at sites 28–33 (where the PWM values are the largest), Q_7_ at sites 28–34, Q_10_ at sites 28–37, Q_11_ at sites 28–38, Q_21_ at sites 18–38, and so on.

**Figure 4:**
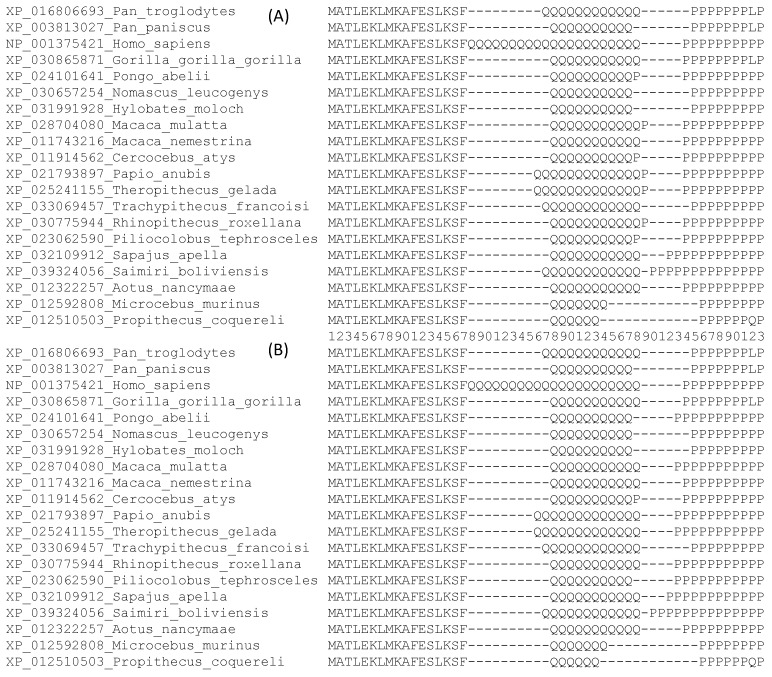
Illustration of PWM-based refinement of sequence alignment based on the N-terminus of 20 aligned HTT sequences, with the site numbering in the middle. (**A**) Alignment after Step 1 refinement. (**B**) Alignment after Step 2 refinement, except that the shared gap at site 39 has not yet been deleted.

The Step 1 refinements result in a favorable PWMD of 9.198. The alignment after Step 1 is shown in Figure 4A.

**Step 2:** The alignment after Step 1 (Figure 4A) has three P residues at site 38 mixed with 12 Q residues. At site 43, there are two P residues without any other amino acids. Moving these three P residues from site 38 in Figure 4A to site 43 increases the PWMD. Similarly, shifting the four P residues from site 39 in Figure 4A to site 43 also increases the PWMD. These refinements result in the alignment in Figure 4B. Such refinements also result in a shared gap at site 39 (Figure 4B) which can be deleted. These refinements yield a further gain of PWMD of 101.4509 (relative to the alignment in Figure 4A).

After Step 2, no further refinement will result in a positive PWMD. Note that the alignment in Figure 4B, after deleting the shared gap at site 39, looks different from that in Figure 3B, but they are equally good based on the PWMD criterion (i.e., changing one to the other will have PWMD = 0). In fact, there are many alternative alignments that are equally good to the alignment in Figure 3B based on the PWMD criterion. They also produce the same tree lnL.

## 4. Discussion

While the PWMD criterion appears promising for post-alignment adjustment, this paper is no more than a proof of concept. There are obviously cases more complicated than the two illustrative examples. Such cases may require multiple iterations of PWM computation and MSA refinement. However, the PWM-based approach does feature three advantages. Firstly, it is conceptually simple. Secondly, it uses information from all sequences. Thirdly, it is fast because PWM requires little computation time, so multiple iterations of refinements can be accomplished in little time.

As I have illustrated, the PWMD criterion can conflict with the SPS and PAI criteria. This is disconcerting given that SPS [22,23,24,25,26], its weighted form [13,21], as well as PAI [17,27,28,29,30,31] have been used frequently both in generating MSAs and in iterative MSA refinement. One may argue that PWMD uses information from all sequences, so it is preferable over the SPS and PAI criteria which are based on information from pairwise alignment and have been criticized in this context [13,21]. It might indeed be time to reconsider SPS and PAI as criteria for MSA refinement.

None of the three criteria illustrated here incorporate the evolutionary history of the aligned sequences. Unfortunately, including an inference of evolutionary history among the sequences in the MSA refining process will invariably demand intensive computation [19,20]. The PWMD criterion, although making use of information from all sequences, implicitly treats all sequences equally as if they were from a star tree. Whether this feature of PWMD might have the benefit of not biasing subsequent phylogenetic estimation would require further studies.

The PWMD criterion has not yet been implemented in any publicly available software for post-alignment adjustment, so its performance has not been explored in any significant scale. Whether it will be adopted by the research community depends on not only the theoretical justification, but also the implementation of the method in user-friendly software packages.

I should emphasize the effect of taxon sampling on sequence alignment and post-alignment adjustment, as this effect is important but often neglected. Take the alignment in Figure 1, for example. The ACE2 sequence in *Mus musculus*, which is not in the alignment, is “LT” at sites 20 and 21. If I remove the first four (primate) ACE2 sequences in Figure 1 and add many ACE2 sequences similar to that of *M. musculus*, then the three post-alignment adjustment approaches would all favor “-T” at sites 20 and 21 against the alternative “T-“ in *N. procyonoides,* contrary to the post-alignment adjustment that we have made before. This again highlights the need to incorporate evolutionary history in post-alignment adjustments. If *M. musculus* is phylogenetically closer to *N. procyonoides* than the first four primate species in Figure 1, then we should keep “-T” at sites 20 and 21 in *N. procyonoides.* In contrast, if the first four primate species are phylogenetically closer to *N. procyonoides,* then we should revise “-T” in *N. procyonoides* (Figure 1) to “T-“. While many researchers have highlighted the effect of taxon sampling on phylogenetic reconstruction [44,45,46], few have so far recognized the fact that the effect of taxon sampling is often seeded in multiple-sequence alignment.

## 5. Conclusions

I illustrated the importance of post-alignment adjustment, outlined criteria to rapidly evaluate alternative alignments, and presented three approaches towards post-alignment adjustment. I highlighted the potential of the position weight matrix approach and illustrated its applications for refining several sets of real sequences. The problem of post-alignment adjustment is not fully solved. I hope that my presentation of the problem and the directions towards a solution will stimulate further research in this rapidly developing field.

## Figures and Tables

**Table 1:** Sum-of-pairs scores for Alignment 1 (Figure 1) and an alternative Alignment 2 with “T-” occupying sites 20 and 21 in *N. procyonoides.* Only sites 20 and 21 in Figure 1 are considered.

	T/-(1)	T/T(1)	T/I(1)	I/-(1)	SPS
Score(2)	−6	5	−1	−6	
Alignment 1	10	6	4		−34 + C(3)
Alignment 2	6	10		4	−10 + C(3)

(1) Amino acid pairs relevant for the calculation of SPS, (2) Gap penalty is −6, T/T match and T/I mismatch scores are 5 and −1, respectively, (3) C is a constant represents sum of pairwise scores from all sequences other than *N. procyonoides*.

**Table 2:** Partial position weight matrix for 11 aligned ACE2 sequences, generated from DAMBE [40] using default options for pseudocounts and background frequencies. Only sites 20 and 21 are included.

	Alignment 1			Alignment 2	
AA	Site 20	Site 21		Site 20	Site21
A	−3.4621	−3.4621		−3.4621	−3.4621
R	−3.4632	−3.4632		−3.4632	−3.4632
N	−3.4620	−3.4620		−3.4620	−3.4620
D	−3.4625	−3.4625		−3.4625	−3.4625
C	−3.4757	−3.4757		−3.4757	−3.4757
Q	−3.4632	−3.4632		−3.4632	−3.4632
E	−3.4616	−3.4616		−3.4616	−3.4616
G	−3.4625	−3.4625		−3.4625	−3.4625
H	−3.4673	−3.4673		−3.4673	−3.4673
I	−3.4628	2.9353		−3.4628	2.9353
L	−3.4612	−3.4612		−3.4612	−3.4612
K	−3.4624	−3.4624		−3.4624	−3.4624
M	−3.4645	−3.4645		−3.4645	−3.4645
F	−3.4629	−3.4629		−3.4629	−3.4629
P	−3.4628	−3.4628		−3.4628	−3.4628
S	−3.4619	−3.4619		−3.4619	−3.4619
T	4.1089	3.5976		4.2457	3.3770
W	−3.4649	−3.4649		−3.4649	−3.4649
Y	−3.4632	−3.4632		−3.4632	−3.4632
V	−3.4621	−3.4621		−3.4621	−3.4621

**Table 3:** Tree log-likelihood values for the two multiple sequence alignments in Figure 3, obtained with PhyML and three different substitution matrices.

	Substitution Matrix		
Alignment	LG	JTT	BLOSUM62
in Figure 3A	−126.6903	−122.6004	−126.7423
in Figure 3B	−106.7703	−105.2280	−106.9387

**Table 4:** Part of the 20 × 3156 position weight matrix obtained with default options for pseudocounts and background frequencies in DAMBE [40]. Only sites 18 to 44 from 3156 aligned sites are shown, with only two amino acids (Q and P) out of 20. Site numbers are as in the alignment in Figure 3A.

Site	Q	P
18	−0.0374	−4.3223
19	−0.0374	−4.3223
20	−0.0374	−4.3223
21	−0.0374	−4.3223
22	−0.0374	−4.3223
23	−0.0374	−4.3223
24	−0.0374	−4.3223
25	1.4974	−4.3223
26	1.4974	−4.3223
27	2.2241	−4.3223
28	4.2125	−4.3223
29	4.2125	−4.3223
30	4.2125	−4.3223
31	4.2125	−4.3223
32	4.2125	−4.3223
33	4.2125	−4.3223
34	4.1387	−4.3223
35	4.0609	−4.3223
36	4.0609	−4.3223
37	4.0609	−4.3223
38	2.8964	2.5727
39	−0.0374	−4.3223
40	−4.3224	−0.1637
41	−4.3224	−0.1637
42	−4.3224	0.7953
43	−4.3224	0.7953
44	0.9251	3.4601

## Data Availability

Not applicable.

## Supplementary Materials

The following are available online at https://www.mdpi.com/article/10.3390/genes12111809/s1, Primate_HTT_MAFFT.fas.
